# Oral Diadochokinesis, Tongue Pressure, and Lip-Seal Strength Among Japanese Male Workers in the Taxi Industry: A Cross-Sectional Study

**DOI:** 10.3390/clinpract14060196

**Published:** 2024-11-14

**Authors:** Akira Minoura, Yoshiaki Ihara, Hirotaka Kato, Kouzou Murakami, Yoshio Watanabe, Kojiro Hirano, Yoshinori Ito, Akatsuki Kokaze

**Affiliations:** 1 Department of Hygiene, Public Health and Preventive Medicine, School of Medicine, Showa University, 1-5-8 Hatanodai, Shinagawa-ku, Tokyo 142-8555, Japan; 2 Division of Oral Functional Rehabilitation Medicine, Department of Special Needs Dentistry, School of Dentistry, Showa University, 2-1-1 Kitasenzoku, Ohta-ku, Tokyo 145-8515, Japan; 3 Department of Radiology, Division of Radiation Oncology, School of Medicine, Showa University, 1-5-8 Hatanodai, Shinagawa-ku, Tokyo 142-8555, Japan; 4 Division of Respiratory Medicine and Allergology, Department of Medicine, School of Medicine, Showa University, 1-5-8 Hatanodai, Shinagawa-ku, Tokyo 142-8555, Japan; 5 Department of Otorhinolaryngology Head and Neck Surgery, School of Medicine, Showa University, 1-5-8 Hatanodai, Shinagawa-ku, Tokyo 142-8555, Japan

**Keywords:** oral diadochokinesis, tongue pressure, lip-seal strength, workers, Japanese

## Abstract

**Background/Objectives:** Health management in workers in the taxi industry is particularly challenging due to irregular working hours and the need to prevent fatal accidents. In addition, drivers in Japan are aging, and the early prevention of age-related deterioration in oral health is an increasingly important issue. The aim of this cross-sectional study was to investigate the relationships between oral diadochokinesis (OD), tongue pressure, and lip-seal strength in Japanese male taxi workers. **Methods:** Measurements of tongue pressure and lip-seal strength were performed by dentists using specialized equipment. OD was measured using the number of consecutive “Pa”, “Ta”, and “Ka” vocalizations that could be produced in 5 s. We performed multiple regression analysis to examine the effects of lip-seal strength and tongue pressure on OD. **Results:** The study included 437 participants, excluding 17 who could not complete all oral cavity measurements. Tongue pressure showed a significant positive correlation with “Pa”, “Ta”, and “Ka” (correlation coefficients: 0.527–0.680). Lip-seal strength was not significantly correlated with OD. Tongue pressure showed a significant positive correlation with “Pa”, “Ta”, and “Ka”. In the results of multiple regression analyses without the elderly participants, tongue pressure was associated with “Pa” (β[95% confidence interval]: 0.574[0.304, 0.843]), “Ta” (0.436[0.231, 0.640]), and “Ka” (0.424[0.210, 0.639]), and lip-seal strength was associated with “Pa” (0.128[0.032, 0.224]) and “Ka” (0.083[0.006, 0.160]). **Conclusions:** OD may be associated with lip-seal strength and tongue pressure even without including elderly workers. Regardless of age, maintaining good OD may help maintain lip-seal strength and tongue pressure, which may play a role in reducing the risk of age-related oral disorders.

## 1. Introduction

Oral diadochokinesis (OD) is a test that evaluates the mobility of the oral organs, including the tongue, lips, and jaw, among the elderly [1,2]. Simultaneously, OD is a typical assessment tool used to evaluate speech motor skills or speech development in children [3,4]. It uses repetitive vocalizations of vowels and consonants to measure the accuracy, speed, and consistency of oral movements [5]. In many previous studies, OD has been assessed by repeated vocalizations of the vowels and consonants of ‘pa’, ‘ta’, and ‘ka’ [1,2,3,4,5]. Dentists and speech and language pathologists primarily perform OD to evaluate movement disorders caused by dysarthria, delayed speech development, and neurological disorders [4]. In contrast, in several neurological disorders, tongue pressure and lip-seal strength may be sensitive indicators of articulatory function and oral motor impairment [5]. Although tongue pressure, lip-seal strength, and OD may be associated with a decline in oral function and sarcopenia in the elderly, how these factors relate to OD in younger and middle-aged individuals remains unclear [1,6]. Tongue pressure and lip-seal strength are associated with alcohol use, smoking habits, and body mass index (BMI) among healthy Japanese individuals [7]. We have shown the trend in tongue pressure and lip-seal strength by age group among Japanese workers in the taxi industry, where health management is especially challenging owing to irregular working hours and the need to prevent fatal accidents [7]. In particular, lip-seal strength was lower in young adults than that in older adults, and tongue pressure was lower in young individuals than in middle-aged adults [7]. Although lip-seal strength is mainly influenced by the muscles surrounding the mouth, declining oral function cannot be solely attributed to aging; lifestyle factors, such as eating habits earlier in life, may affect its intensity. Measuring tongue pressure and lip-seal strength requires a special instrument; however, OD does not require special equipment and is easy to measure; therefore, investigating the relationship between tongue pressure, lip-seal strength, and OD can lead to a simple measurement of the oral environment or prevent oral disorders [6,8].

Previous studies have reported that enhancing oral health may prevent daytime sleepiness due to obstructive sleep apnea [9,10]. Our previous study demonstrated that maintaining moderate lip-seal strength prevents daytime sleepiness in workers [11]. OD could help reduce sleep-related accidents among workers, particularly the elderly, at a cheap cost because the Japanese government is encouraging the employment of elderly citizens, the percentage of whom is rapidly rising in the country [12]. Because obstructive sleep apnea is believed to be intensified by age-related tongue root depression, OD improvement may help in avoiding daytime sleepiness [11]. Particularly in jobs requiring life-threatening duties, including working as a security guard or driver, measuring OD is crucial in helping to forecast excessive daytime sleepiness. In addition, although previous studies have examined OD in healthy adults, they have not examined OD in workers, nor have they examined the effects of lifestyle factors such as alcohol consumption and smoking [8]. Therefore, examining the relationship among OD, lip-seal strength, and tongue pressure in younger age groups has implications for understanding the prevention of declined oral function and sarcopenia in workers. We hypothesized that tongue pressure and lip-seal strength would be significantly associated with OD and that these associations may vary with age and occupational characteristics.

The aim of this work is to examine the correlation between tongue pressure, lip-seal strength, and OD across different age groups with the aim of preventing a decline in oral function and sarcopenia among Japanese male workers in the taxi industry.

## 2. Materials and Methods

### 2.1. Study Design

This study was conducted between November 2021 and June 2022 and involved 454 male employees from two Japanese taxi companies (the recruitment period was from 9 November 2021 to 22 June 2022). The exclusion criteria included those with a history of cerebrovascular disease, neuromuscular disease, cranial cervical tumors, etc., and those who had difficulty maintaining posture during recording. After excluding 17 participants who met this exclusion criterion, the analyses in this study included 437 participants.

### 2.2. Measurements

An experienced dentist instructed the study participants in the OD task measurement and recorded the results. The instruction for participants was “Please repeat the syllables ‘pa’, ‘ta’, and ‘ka’” as quickly as possible for 5 s. This instruction was based on previous studies [9,10]. All participants were seated during the measurement, and the number of repeated syllables was recorded. Several previous studies conducted in other countries have used similar monosyllabic test sounds, and we also considered this method to be effective, regardless of the native language [10,13,14]. Furthermore, we trained a dentist to measure lip-seal strength [N] and tongue pressure [kPa] in all participants. We used a reliable specialized device (Lipple Kun, Shofu, Kyoto, Japan) to measure the lip-seal strength [11,12,13,14]. We measured tongue pressure using specialized equipment (TPM-01; JMS Co., Ltd., Hiroshima, Japan). We determined the participants’ tongue pressure by averaging the three recordings. We took each measurement after a minimum of 1 min of rest following the previous measurement [15]. The measurement methods and details of the devices with images can be found in our previous study [11].

### 2.3. Covariates

We used a self-administered questionnaire with items on age, alcohol use (once a week or more, less than once a week), smoking habits (yes, no), weight (kg), and height (cm), because studies have associated alcohol use, smoking, and overweight/obesity with tongue pressure and lip-seal strength among healthy Japanese individuals [7,15]. The COVID-19 pandemic in Japan has led to changes in job descriptions, such as from drivers to clerks, which we did not include in the questionnaire.

### 2.4. Statistical Analysis

We used the Shapiro–Wilk test for continuous factors to verify a normal contribution. This study displays median values, specifically the 25th and 75th percentiles, for continuous variables with non-normal distribution. We computed correlation coefficients (Spearman’s r) before performing analysis to verify multicollinearity between tongue pressure, OD, lip-seal strength, and other variables. *p*-values < 0.05 were used to denote statistical significance. In the primary statistical analysis, we investigated the age-group-specific relationships between tongue pressure, lip-seal strength, and OD using multiple regression analysis. We have presented the data along with 95% confidence intervals. This study adhered to the Strengthening the Reporting of Observational Studies in Epidemiology standard for conducting cross-sectional studies. Statistical analyses were performed using JMP (version 17.0; SAS Institute, Inc., Cary, NC, USA).

## 3. Results

In this study, to examine the characteristics of OD by age, we stratified the 437 participants into those in their 20 s, 30 s, 40 s, 50 s, and >60 s. This study excluded participants under the age of 20 years. The characteristics of the study participants are shown in Table 1.

Table 2 shows the correlation coefficients between OD and covariates, excluding workers aged ≥60 years. The results indicate a significant positive correlation between “Pa”, “Ta”, and “Ka”, with correlation coefficients of 0.527–0.680. Tongue pressure had a significant positive correlation with “Pa”, “Ta”, and “Ka”, although lip-seal strength had a nonsignificant positive correlation. No significant relationship was observed between BMI and “Pa”, “Ta”, and “Ka”. Furthermore, “Ka” showed a significant negative correlation with age. Upon evaluation, we determined that none of the factors strongly correlated with any of the other covariates. To prevent multicollinearity, we decided to incorporate all factors into the analysis.

Table 3 presents a concise overview of the relationship between lip-seal strength, tongue pressure, and OD across different age groups. We conducted several regression models to separately investigate the effects of lip-seal strength and tongue pressure on OD. Model 1 found a significant correlation between “Pa”, “Ta”, and “Ka” and tongue pressure. Model 2 found a significant correlation between “Pa” and “Ka” and lip-seal strength. We adjusted both models for age, BMI, alcohol consumption, and smoking.

## 4. Discussion

This appears to be the first investigation into the correlation between OD and specific lip-seal strength and tongue pressure among Japanese male workers in the taxi industry. After adjusting for age, BMI, alcohol consumption, and smoking, the results showed a positive correlation between tongue pressure and “Pa”, “Ta”, and “Ka”. Moreover, lip-seal strength showed a positive correlation with “Pa” and “Ka”.

These results indicate that regardless of age, maintaining good OD contributes to preserving lip-seal strength and tongue pressure, potentially playing a role in reducing the risk of oral disorders associated with aging [6,15]. The study results revealed that OD had mostly been used to evaluate frailty and oral diseases in older individuals, at least in Japanese studies before this one [1]. Although this cross-sectional study cannot examine causality between OD and health-related outcomes, it can be used in future longitudinal studies to determine health-related outcomes among younger workers [1,16,17]. Moreover, a previous study had shown that daytime sleepiness is a serious risk factor for insulin resistance [18]. Regardless of age or weight, daytime sleepiness may contribute to the development of insulin resistance [19]. Although we demonstrated that tongue pressure and lip-seal strength are associated with daytime sleepiness in our previous study, OD may also be associated with daytime sleepiness [11]. Future research should examine causal relationships among eating patterns, daytime sleepiness, and oral environment markers, including lip-seal strength and tongue pressure. Training perioral muscle function may improve percutaneous oxygen saturation during sleep, despite reports linking it to sleep disturbances and diabetes mellitus (DM) [20]. Interventions can help modify eating and OD behaviors, which could prevent DM and daytime sleepiness from sleep apnea [11,20]. For workers, sleep problems can lead to serious health problems associated with excessive daytime sleepiness, including hypertension and cerebral vascular disease [21]. Considering Japan’s aging population and falling birth rate, protecting the labor force by preventing or treating sleep disorders early is imperative. The outcomes of this study may contribute to the development of novel approaches to support OD-based sleep disorder prevention or early intervention. In Japan, some reports have suggested a link between tongue pressure and obstructive sleep apnea [22]. We chose to identify and evaluate the OD of workers based on oral health examinations because the absence of subjective symptoms raised the possibility that many patients with sleep disorders may go unnoticed. As a consistent finding, tongue pressure and lip-seal strength were reportedly reduced in older adults [7]. Although the muscle activities in OD, tongue pressure, and lip-seal strength have been proven to be reduced in elderly people, only a few previous studies have been conducted on younger age groups, which represents a strength of this study [23]. Previous studies noted that the lip-seal strength is mainly influenced by the muscles surrounding the mouth (e.g., the orbicularisoris), which are stronger in young adults than in older adults [8,24]. Other studies have also reported that tongue pressure values tend to be low in patients with low OD values. Tongue pressure was reported to be significantly lower in those who were able to say “Ka” less than four times than in those who were able to say it four or more times in OD, and our study indicates similar results [14]. A possible positive correlation between tongue pressure and OD has also been reported in healthy adults [25]. This may be related to the fact that OD is performed to evaluate the skill and speed of lingual movement, and a sound such as “Ta” is produced by the contact between the tongue and palate. “Ta” and “Ka” perform a similar movement in that the tongue is elevated toward the palate, which may be one of the factors contributing to the positive correlation between OD results and tongue pressure.

There were several limitations to this study. First, we were unable to examine causal relationships between tongue pressure, lip-seal strength, and OD because this study was based on a cross-sectional survey. Second, although some research has suggested sex differences in lip-seal strength and OD, these differences could not be examined in the current study [26,27]. Based on our study results on male workers, future studies should include and discuss female workers. Furthermore, because the Japanese taxi industry tends to have more male than female workers, surveys should be conducted in various industries in the future. Third, in this study, evaluating how the COVID-19 epidemic affected the participants’ dental-health-related lifestyle habits was impossible. Changes in the lifestyle (particularly in terms of BMI and eating habits) of workers caused by COVID-19 preventative initiatives may have impacted the measurement of OD [28,29]. Future longitudinal studies will be necessary to accurately assess the pandemic’s impact. Finally, as this study did not include sufficient data for social and physical contexts among workers, the reference values for the OD test to indicate functional impairment vary and are controversial [30].

## 5. Conclusions

We found that tongue pressure and lip-seal strength may be associated with OD even without including elderly workers. Our findings indicate that regardless of age, maintaining good OD helps preserve lip-seal strength and tongue pressure, potentially reducing the risk of age-related oral disorders.

## Figures and Tables

**Table 1:** Characteristics of the study participants by age group.

	Total	20 s	30 s	40 s	50 s	60 s and Over	^1)^ *p*-Value
	(N = 437)	(n = 85)	(n = 63)	(n = 87)	(n = 143)	(n = 59)	
Lip-seal strength [N]	13.8 (11.6, 16.4)	12.1 (9.6, 14.0)	12.9 (11.6, 15.8) *	14.7 (12.4, 17.3) *	14.3 (12.2, 16.9) *	14.9 (12.0, 18.1) *	<0.001
Tongue pressure [kPa]	42.1 (35.9, 48.9)	40.9 (33.5, 48.1)	45.5 (37.7, 50.1)	44.5 (35.3, 50.2)	42.0 (36.7, 47.5)	41.0 (34.9, 46.7)	0.071
Age [years]	47.0 (31.0, 55.0)						
BMI [kg/m^2^]	24.1 (21.6, 27.2)	21.8 (19.8, 24.6)	24.1 (22.4, 28.3) *	24.6 (22.3, 27.7) *	24.3 (22.2, 27.4) *	25.1 (23.0, 28.7) *	<0.001
Pa [times]	33 (31, 35)	34 (30, 36)	34 (32, 36)	34 (32, 36)	33 (31, 34)	32 (29, 33) *	<0.001
Ta [times]	35 (32, 38)	36 (33, 39)	35 (33, 40)	36 (33, 39)	35 (32, 37)	32 (30, 34) *	<0.001
Ka [times]	32 (29, 34)	33 (30, 35)	32 (30, 36)	32 (29, 36)	31 (29, 33)	29 (26, 32) *	<0.001
Alcohol use [once a week or more]	259 (59.3)	46 (54.1)	34 (54.0)	56(64.4)	82 (57.3)	41 (69.5)	0.251
Smoking [yes]	163 (37.3)	25 (29.4)	23 (36.5)	44 (50.6)	51 (35.7)	20 (33.9)	0.054

Except where indicated n (%), values are median (25th, 75th percentile). BMI: Body mass index. ^1)^ Analysis of variance was performed for continuous variables, and the Chi-square test was performed for dichotomous variables. * The results of Dunnett’s test by age, using participants in their 20 s as controls, were significantly lower or higher.

**Table 2:** Correlation coefficients between oral diadochokinesis and covariates (excluding 60s and over).

				Spearman’s r			
	a	b	c	d	e	f	g
a. Pa		0.613 *	0.527 *	0.094	0.184 *	−0.045	−0.052
b. Ta			0.680 *	0.073	0.201 *	−0.099	0.015
c. Ka				0.061	0.189 *	−0.101 *	−0.029
d. Lip-seal strength					0.396 *	0.278 *	0.288 *
e. Tongue pressure						0.080	0.274 *
f. Age							0.178 *
g. BMI							

Values are correlation coefficients. BMI: Body mass index. * *p* < 0.05.

**Table 3:** Associations between tongue pressure, lip-seal strength, and oral diadochokinesis (results of multiple regression analyses).

	Pa		Ta		Ka	
	Model 1	Model 2	Model 1	Model 2	Model 1	Model 2
Tongue pressure	0.574 (0.304, 0.843) *		0.436 (0.231, 0.640) *		0.424 (0.210, 0.639) *	
Lip-seal strength		0.128 (0.032, 0.224) *		0.072 (−0.001, 0.145)		0.083 (0.006, 0.160) *
Age	0.027 (−0.052, 0.107)	0.069 (0.041, 0.097) *	0.038 (−0.042, 0.118)	0.073 (0.044, 0.101) *	0.029 (−0.051, 0.110)	0.072 (0.043, 0.101) *
BMI	0.634 (0.419, 0.848) *	0.203 (0.127, 0.280) *	0.578 (0.364, 0.793) *	0.191 (0.115, 0.268) *	0.590 (0.374, 0.805) *	0.198 (0.121, 0.276) *
Alcohol use	−0.062 (−0.994, 0.871)	−0.130 (−0.462, 0.201)	0.118 (−0.812, 1.048)	−0.103 (−0.434, 0.229)	−0.133 (−1.066, 0.801)	−0.124 (−0.458, 0.211)
Smoking	−0.314 (−1.267, 0.639)	−0.263 (−0.602, 0.076)	−0.281 (−1.230, 0.667)	−0.255 (−0.593, 0.084)	−0.327 (−1.280, 0.626)	−0.277 (−0.618, 0.064)

Values are standardized beta (95% confidence interval). BMI: Body mass index. Model 1: Association between oral diadochokinesis and tongue pressure adjusted for age, BMI, alcohol use, and smoking. Model 2: Association between oral diadochokinesis and lip-seal strength adjusted for age, BMI, alcohol use, and smoking. * *p* < 0.05.

## Data Availability

In this study, the data are not available in a public repository but are available from the corresponding author upon reasonable request (s001050@gmail.com).
